# Unilateral morning glory optic disc anomaly in a case with Down syndrome

**DOI:** 10.1186/1471-2415-14-48

**Published:** 2014-04-13

**Authors:** Ahmet Altun, Gulengul Altun, Sevda Aydin Kurna, Osman Okan Olcaysu, Suat Fazil Aki

**Affiliations:** 1 Fatih Sultan Mehmet Education and Research Hospital, Clinic of Ophthalmology, Istanbul, Turkey; 2 Department of Pediatrics, Yeditepe University, Istanbul, Turkey; 3 Erzurum Region Education and Research Hospital, Clinic of Ophthalmology, Erzurum, Turkey

**Keywords:** Morning glory, Optic disc anomaly, Down syndrome

## Abstract

**Background:**

This case is unique because it is the first reported case of Down syndrome with morning glory optic disc anomaly in literature.

**Case presentation:**

A 15-year-old girl with features of Down syndrome presented to the Clinic of Ophthalmology for a regular ophthalmologic examination. Her best corrected visual acuity was 20/50 in the right eye and 20/20 in the left eye. The fundus examination revealed findings compatible with unilateral morning glory optic disc anomaly in the right eye. The patient underwent a complete ophthalmologic and systemic evaluation to explore possible associated findings.

**Conclusion:**

This case report emphasizes the importance of ophthalmic screening-examinations in Down children to rule out any vision relevant pathology.

## Background

Down syndrome (DS), that was first described by Down in 1866 [1], is the most commonly reported chromosomal abnormality in humans, with an incidence in the United States of one per seven-hundred and thirty-three live births [2]. Various systemic and ophthalmic features found in patients with DS have been reported in the literature [3,4].

Morning glory syndrome (MGS) is an uncommon and generally unilateral congenital anomaly consisting of a funnel-shaped excavation of the posterior pole involving the optic disc. This excavation is usually filled with a white tuft of glial tissue, and surrounded with a pigment ring slightly protruding into the peripapillary zone. The number of retinal vessels is multiplied, and they appear to arise and tend to run to the peripheral retina in a radial course. The anomaly was named ‘morning glory syndrome’ because of its similarity in appearance to the tropical morning glory flower, and firstly described in 1970 by Kindler [5].

In this report we would like to present unilateral MGS in a case with DS. This report is unique because it is the first reported case of DS has MGS in the literature.

## Case presentation

**Figure 1:**
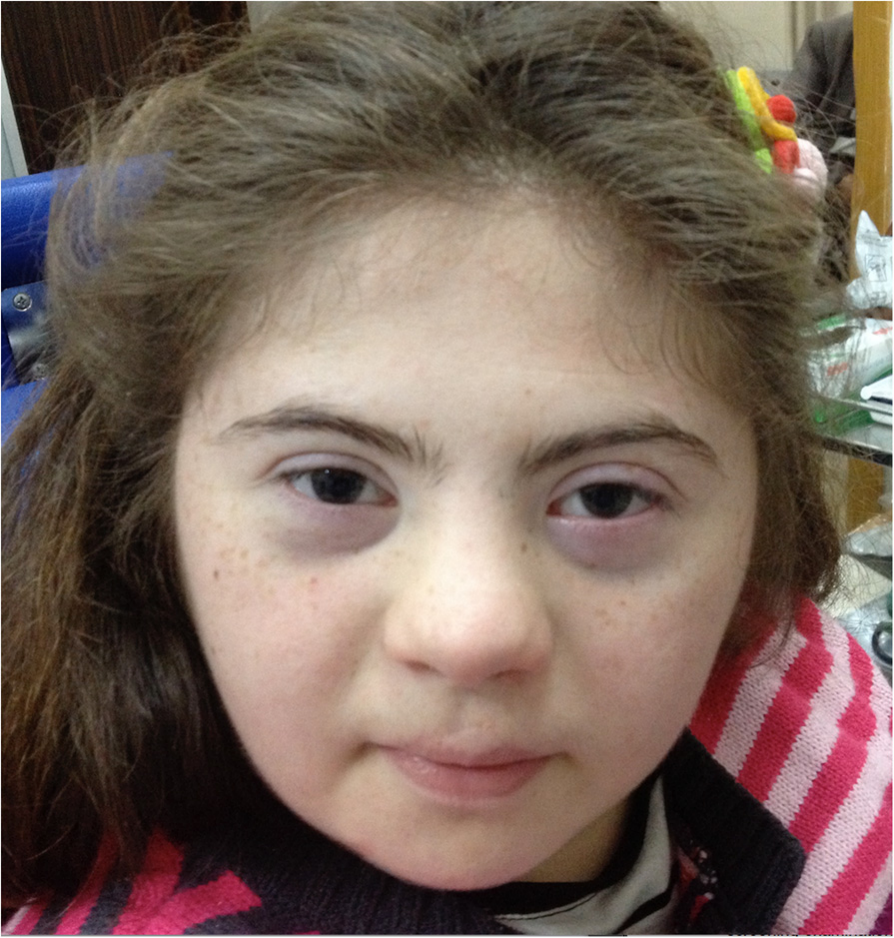
Picture of the case with the characteristic face of Down syndrome.

**Figure 2:**
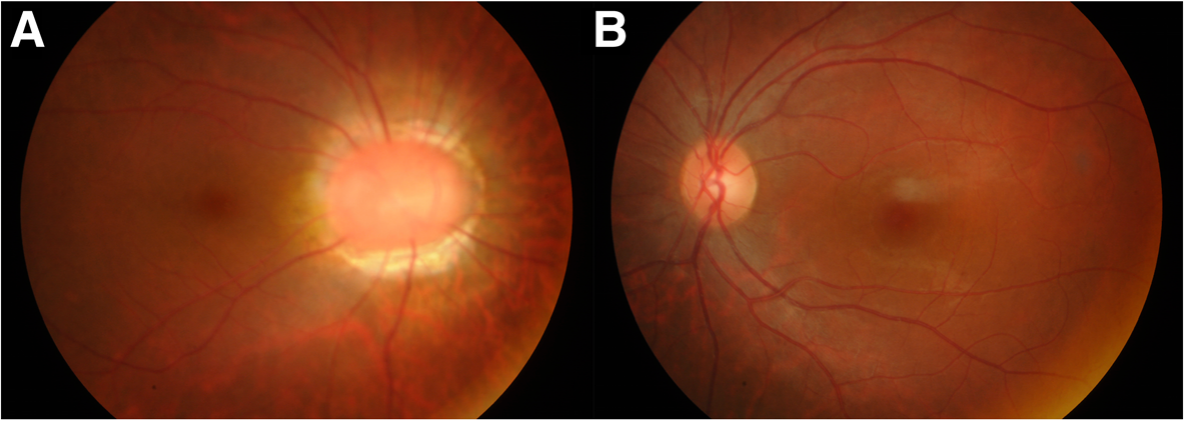
Fundus photographs of the right eye (A) and the left eye (B).

A 15-year-old girl with the characteristic face of Down syndrome (DS), who had previously documented trisomy 21, referred to the Clinic of Ophthalmology of Fatih Sultan Mehmet Education and Research Hospital (Figure 1). The funduscopic examination of the right eye revealed a funnel-shaped optic disc with a central glial tuft and thin radiating retinal vessels emerging at the optic margin (Figure 2A), accordant with MGS and no abnormality in the left eye. (Figure 2B). The patient underwent a complete ophthalmologic and systemic evaluation to explore associated findings.

Her mental status was adequate to communicate without trouble. Her best corrected visual acuity was 20/50 in the right eye and 20/20 in the left eye. Her best corrected near vision was J3 in the right eye and J1+ in the left eye according to Jaeger. Anterior segment examination and intraocular pressures were unremarkable and were within normal limits (Right eye: 13 mmHg, Left eye: 15 mmHg). There was a mild relative afferent pupillary defect in the right eye. There were isocoria and normal pupillary near responses. Ocular alignment was orthophoric, and extraocular muscle movement was not limited. Refraction examination revealed myopia of -3,00 D in the right eye. Corneal topography revealed a normal pattern excluding keratoconus. There was no refraction error in the left eye. Axial length was 24.35 mm in the right eye and 22.54 mm in the left eye. Keratometric values of K1/K2 were 41,25/41,50 D in the right eye and 41.00/41.50 D in the left eye.

**Figure 3:**
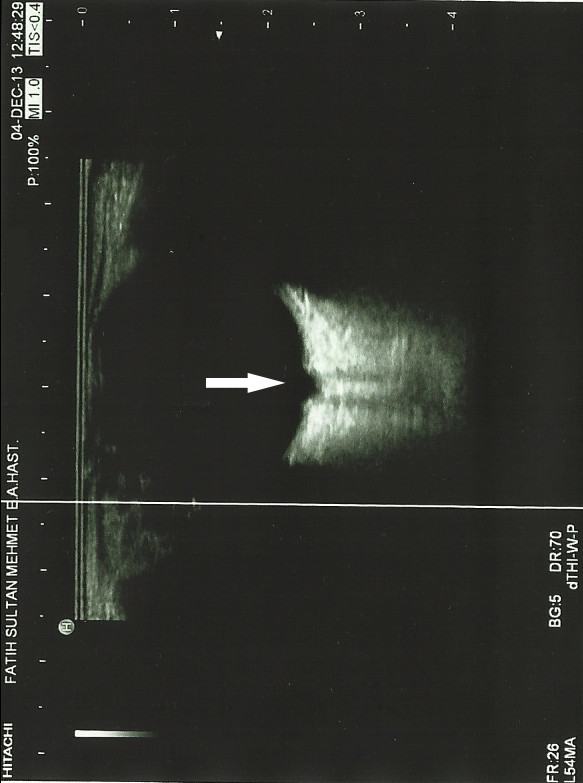
Conoid excavation in the posterior pole with the optic disc in the right eye.

B-scan ultrasonography showed a conoid excavation in the posterior pole with the optic disc in the base (Figure 3), where as the posterior pole of the left eye was normal. Magnetic resonance imaging (MRI, 3-tesla) of the cranium and orbita were within normal limits. There was no encephalocele, agenesia of corpus callosum, asymmetry of optic nerve sheaths, or abnormality of carotid circulation bilaterally.

**Figure 4:**
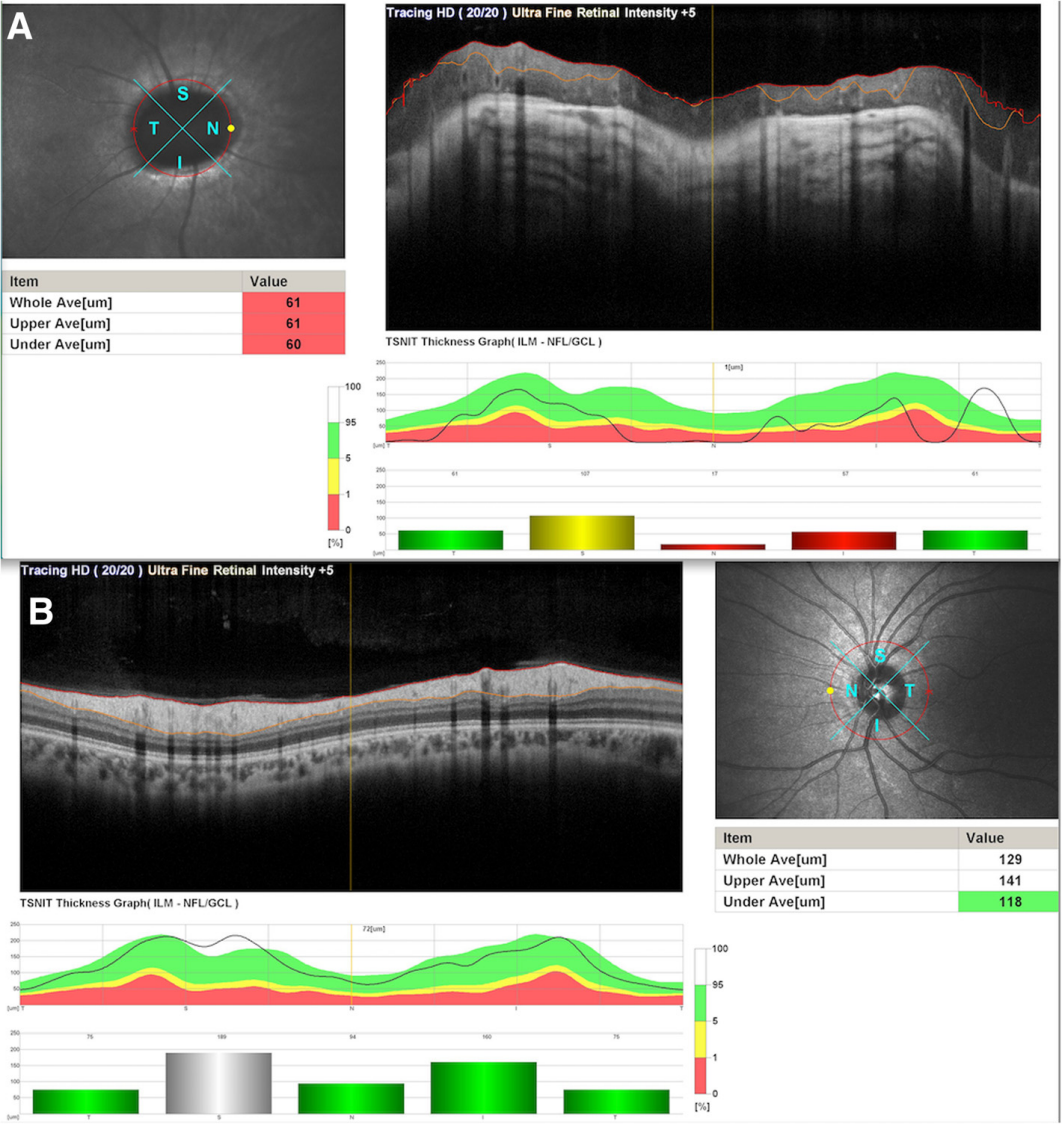
Retinal nerve fiber layer was reduced in the right eye (A) and within the normal limits in the left eye (B).

**Figure 5:**
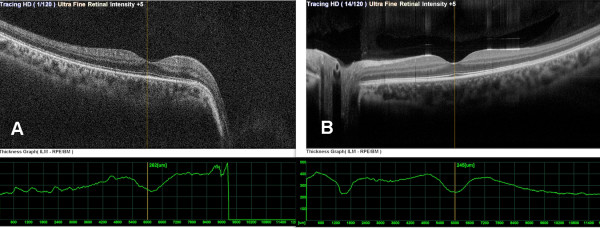
Axial optical coherence tomography scan of macula of the right eye (A) and the left eye (B).

**Figure 6:**
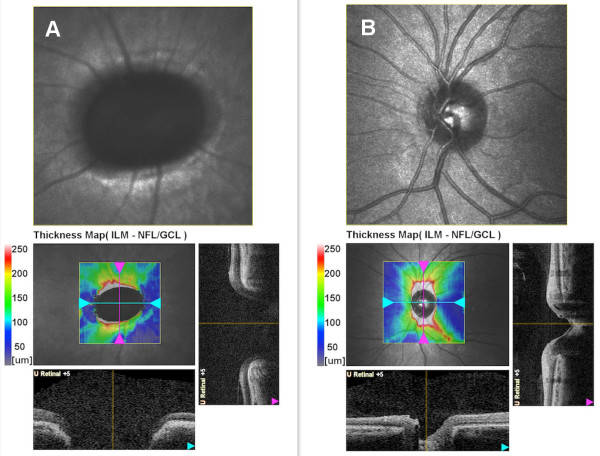
Coronal and sagittal optical coherence tomography scan of the optic nerve head of the right eye (A) and the left eye (B).

**Figure 7:**
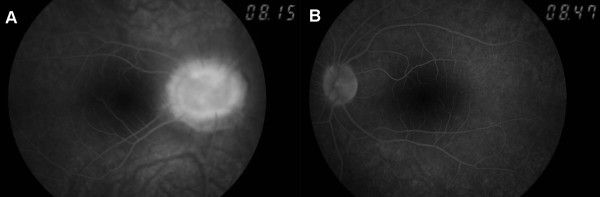
Late phase of the fundus fluorescein angiography of the right eye (A) and the left eye (B).

Spectral domain optical coherence tomography (SD-OCT) (Nidek, RS-3000, Japan) revealed reduced retinal nerve fiber layer and glial tissue in the center of the excavation (Figure 4A) in the right eye, where as it was within normal limits in the left eye (Figure 4B). There was no determined break, contraction, or subretinal fluid in the posterior pole (Figure 5 and Figure 6), and that was confirmed by fundus fluorescein fundus angiography (Figure 7).

The patient had no family history of any hormonal disorders or congenital anomalies. According to the information obtained from her parents, she was delivered vaginally at 40 weeks of gestation to a mother aged 34. Birth weight 3200 g, cephalic diameter 33 cm, and length 45 cm. She was clinically diagnosed at birth as DS and her karyotype was 47,XX,+21.

## Conclusions

DS is due to trisomy of the whole or part of chromosome 21 in all or some cells of the body and is associated with mental retardation, congenital heart defects, gastrointestinal anomalies, reduced neuromuscular tone, dysmorphic features of the head, neck and airways, characteristic facial and physical features, audiovestibular and visual impairment and a higher incidence of other clinical disorders [6,7]. Our case had documented trisomy 21 previously.

The MGS is a non-hereditary and usually unilateral optic nerve disease demonstrating increased excavation, neuroretinal ring hypopigmentation, radial retinal vessels with glial tissue and a funnel-shaped arrangement [5]. MGS can be confused with peripapillary staphyloma, which unlike MGS, there are no vascular anomalies and central glial tuft [8]. Our patient’s optic disc in her right eye was demonstrating all characteristics of MGS.

DS has been reported to be associated with various ocular findings, such as upslanting palpebral fissures, Brushfield spots, epicanthic folds, and ocular abnormalities with important impact on vision, such as malformation of the eyelids, cornea, iris, and crystalline lens in addition to ametropia, strabismus, nystagmus, glaucoma, optic nerve coloboma, and amblyopia [3,9]. In this report we would like to present unilateral MGS in a case with DS.

Optic disc and optic disc vessels anomalies have been described in association with DS [10-12]. Number of retinal vessels crossing the disc optic margin in the patients with DS is increased (40%) when compared to a normal population (6%) [10]. Sherk et al. reported this condition was true whether or not the DS group had congenital heart defects [11]. Parsa et al. reported supranumery optic vessels might indicate reduced systemic angiogenesis in DS [12]. Our case had supranumery retinal vessels in the right eye and a funnel-shaped optic disc with a central glial tuft accordant to MGS.

MGS usually is more frequent in women, as in our case [8]. Most cases are unilateral [13] but there are rare cases of bilaterality [14]. The etiology of this syndrome is still unknown [15]. It is presumed to be due to an insufficient closure of the embryonic fissure, and therefore may be a variant of optic nerve coloboma, or it may be a primary mesenchymal abnormality [16,17]. More recent studies have suggested that MGS is a primary mesenchymal abnormality resulting in faulty closure of the posterior scleral wall and the poor development of the lamina cribrosa [18].

MGS might be noncontractile or, it might be associated with contractile movements of the optic disc [8,16]. In a recent case report, spontaneous, regular and rhythmic contractions have been shown with a video camera through the teaching mirror attached to the indirect ophthalmoscope [19]. Our case had noncontractile MGS.

Approximately, 38% of the cases with MGS already have retinal detachment at the time of MGS diagnosis [20]. The two likely sources of serous fluid are the cerebrospinal fluid [21] or the vitreous cavity, [22] and tractional forces may have a contribution [23]. Sclera or retinal deficiency may let the fluid leak under the retina. But the origin of the fluid sometimes remains debate. In our case, there was no subretinal fluid or retinal break that may increase the risk of retinal detachment. OCT is very helpful technique to evaluate possible subretinal fluid in early stage, and it may also provide information about the pathogenesis and clinical features of MGS [17,24]. In our case there was no evidence of retinal break or subretinal fluid.

MGS may be associated with hormonal deficiencies and anterior segment dysgenesia more often in bilateral cases. The hypothalamic structures involvement may be associated to endocrine alterations [25]. The pituitary dysfunction could either be due to primary agenesis of the pituitary gland or direct compression of the pituitary gland by the meningoencephalocele. In MGS, deficiencies of growth hormone or anti-diuretic hormone are the most frequent findings in hormonal evaluation [26]. Our case had only secondary hypothyroidism due to deficiency of thyroid-stimulating hormone. There was no determined agenesis of pituitary gland or encephalocele in the cranial MRI.

The MGS usually has an early diagnosis due to poor visual acuity. Reduced vision might be due to the presence of retinal abnormalities or amblyopia secondary to anisometropia or strabismus. The BCVA of the right eye in our patient was 20/50 with the correction of -3,00 D myopia. There was no refractive error in the left eye. She had relative afferent pupillary defect in the right eye. The most likely causes of low vision in the right eye were probably due to anisomotropic amblyopia and possible damage in the optic nerve.

Trans-sphenoidal and sphenoethmoidal encephaloceles in association with MGS have been reported in the literature [13,26]. MGS has been also associated with intracranial vascular abnormalities in as many as 45% of cases [27], such as narrowing or aplasia of the Circle of Willis and agenesis of the internal carotid artery. MRI exam plays a very important role to evaluate extensions of these anomalies [25]. In our case, MRI of the cranium and orbita were within normal limits, there was no encephalocele or agenesia of corpus callosum.

Descriptions of ocular anomalies associated with MGS are numerous, such as ciliary body cyst, aniridia, lens coloboma, strabismus, congenital cataract, nystagmus, eyelid hemangioma, lenticonus, and microphthalmia [5,28-31]. There have been also case reports of miscellaneous associations with MGS, such as hypertelorism, cleft lip and palate, renal anomalies, corpus callosum agenesis, and encephaloceles [22,26]. Our case is unique because this is the first reported case of DS with MGS in literature.

Genetic predisposition to MGS still remains unclear. Mutations in gene PAX6 had been associated with MGS [32,33]. Midline craniofacial defects [13], CHARGE syndrome [34], 47XYY syndrome [35], and neurofibromatosis type 2 [36] have been also reported in association with MGS. The only genetic abnormality in our case was the trisomy of chromosome 21.

In the present case, morning glory optic disc anomaly may be an uncommon manifestation of trisomy 21, or could be coincidence. This case report also emphasizes the importance of ophthalmic screening-examinations in Down children to rule out any vision relevant pathology.

## Consent

Written Parental informed consent was obtained from the parents’ patient for publication of this case report and any accompanying images. A copy of the written Parental consent is available for review by the Editor of this journal.

## Competing interests

The authors declare that they have no competing interests.

## Authors’ contributions

AA and GA have made substantial contributions to analysis and interpretation of data; have been involved in drafting the manuscript or revising it critically for important intellectual content; and have given final approval of the version to be published. OOO, SAK and SFA have been involved in drafting the manuscript or revising it critically for important intellectual content; and have given final approval of the version to be published.

## Pre-publication history

The pre-publication history for this paper can be accessed here:

http://www.biomedcentral.com/1471-2415/14/48/prepub
